# Interactions of NADP-Reducing Enzymes Across Varying Environmental Conditions: A Model of Biological Complexity

**DOI:** 10.1534/g3.112.003715

**Published:** 2012-12-01

**Authors:** Teresa Z. Rzezniczak, Thomas J. S. Merritt

**Affiliations:** Department of Chemistry and Biochemistry, Laurentian University, Sudbury, Ontario, P3E 2C6, Canada

**Keywords:** NADPH, metabolic networks, stress, *Drosophila melanogaster*

## Abstract

Interactions across biological networks are often quantified under a single set of conditions; however, cellular behaviors are dynamic and interactions can be expected to change in response to molecular context and environment. To determine the consistency of network interactions, we examined the enzyme network responsible for the reduction of nicotinamide adenine dinucleotide phosphate (NADP) to NADPH across three different conditions: oxidative stress, starvation, and desiccation. Synthetic, activity-variant alleles were used in *Drosophila melanogaster* for *glucose-6-phosphate dehydrogenase* (*G6pd*), cytosolic *isocitrate dehydrogenase* (*Idh*), and cytosolic *malic enzyme* (*Men*) along with seven different genetic backgrounds to lend biological relevance to the data. The responses of the NADP-reducing enzymes and two downstream phenotypes (lipid and glycogen concentration) were compared between the control and stress conditions. In general, responses in NADP-reducing enzymes were greater under conditions of oxidative stress, likely due to an increased demand for NADPH. Interactions between the enzymes were altered by environmental stress in directions and magnitudes that are consistent with differential contributions of the different enzymes to the NADPH pool: the contributions of G6PD and IDH seem to be accentuated by oxidative stress, and MEN by starvation. Overall, we find that biological network interactions are strongly influenced by environmental conditions, underscoring the importance of examining networks as dynamic entities.

The constituents of living cells do not act in isolation; rather, they are densely connected, forming networks in which individual members interact and influence the concentrations and activities of one another. It is becoming clearer that proteins, such as enzymes, transcription factors, *etc*., need to be considered within the context of networks, not as components in isolation (Lehner *et al.* 2006; Boone *et al.* 2007; Costanzo *et al.* 2010). In addition, the way in which network members interact, or whether they interact at all, may be expected to change across molecular contexts (*i.e.*, cellular conditions, genetic backgrounds) or environmental stressors (Dworkin *et al.* 2009). To account for, or quantify, this variability and cross-interaction, several studies have taken large-scale approaches to quantify how interactions change across conditions (Luscombe *et al.* 2004; Mani *et al.* 2008; Wang *et al.* 2009; Bandyopadhyay *et al.* 2010), but these studies may be limited by the sheer size and complexity of the networks they investigate, making it difficult to obtain direct measures of genetic interactions, to detect smaller-scale interactions, or to examine interactions across a number of different contexts, such as multiple environmental conditions or genetic backgrounds.

Research on relatively small, well-characterized metabolic networks can complement large-scale studies and offer additional insight into consistency of biological networks by allowing for a fine-scale examination across multiple conditions. The nicotinamide adenine dinucleotide phosphate (NADPH) enzyme network in *Drosophila melanogaster* is an example of such a relatively discrete network. This well-studied metabolic network consists of four key enzymes responsible for the reduction of NADP to NADPH: cytosolic isocitrate dehydrogenase (IDH), cytosolic malic enzyme (MEN), and the two oxidative enzymes of the pentose phosphate pathway (PPP), glucose-6-phosphate dehydrogenase (G6PD) and 6-phosphogluconate dehydrogenase (6PGD). Despite their independent functions, studies have shown that the activities of these enzymes are co-regulated, likely due to their shared function in maintaining cellular pools of NADPH for use in a number of downstream processes, such as lipogenesis, antioxidation, and immune response (Geer *et al.* 1976; Wilton *et al.* 1982; Bentley *et al.* 1983; Merritt *et al.* 2005, 2009).

Recent studies of this network (Merritt *et al.* 2005, 2009) quantified the impact of synthetic genetic variation in *G6pd*, *Idh*, and *Men* on the activities of one another and on lipid concentration in adult *D. melanogaster*. These studies found significant interactions among IDH, MEN, and G6PD [measured to represent both NADP-reducing PPP enzymes as per Wilton *et al.* (1982)], although the magnitude and directionality of the change differed depending on which gene was modified and the genetic background. In most cases, the responses documented were compensatory (*e.g.*, reduction in one enzyme was accompanied by increases in another) and support the theory of the enzymes interacting to maintain a cellular pool of NADPH. However, in some situations, the responses were counterintuitive and would appear to exacerbate the loss of NADPH. Lowered IDH activity, for example, was found to be associated with lower MEN activity (Merritt *et al.* 2009). This parallel reduction in activity suggests that the mechanisms controlling the interactions within the NADPH network may be more complex than simple compensation to maintain the NADPH/NADP balance, making this metabolic network experimentally manageable while still including complex interactions.

The cellular demand for NADPH is hypothesized to change with environmental conditions. Under oxidative stress, a condition driven by an increase in the cellular concentration of damaging reactive oxygen species (ROS), NADPH provides the reducing power for a number of enzymatic and small-molecule antioxidants, which work to detoxify ROS [reviewed in Pollak *et al.* (2007), Ying (2008), and Agledal *et al.* (2010)]. As a result, cellular demand for NADPH is expected to be high. On the other hand, during starvation or desiccation, cellular demand for NADPH changes because lipogenesis, which consumes NADPH, is at an absolute minimum and substrate availability for the NADP-reducing enzymes changes due to downregulation of the PPP (Matzkin and Markow 2009).

Here we report our examination of the consistency of metabolic interactions between the NADP-reducing enzymes and two downstream phenotypes (lipids and glycogen content) under oxidative stress, starvation, and desiccation in *D. melanogaster*. Enzyme activity-variant flies, mimicking natural variation seen in wild flies, were used for *G6pd*, *Idh*, and *Men* under each of the conditions in order to quantify the response (or interaction) in other enzymes as well as the two downstream phenotypes. We find that, in general, stress conditions amplify the interactions between NADPH network members and can also change the directionality of the response. Overall, these results highlight the dynamic nature of genetic interactions and emphasize the importance of examining interactions across multiple conditions.

## Materials and Methods

### Fly stocks and lines

Seven isothird-chromosome *D. melanogaster* lines were created from isofemale lines collected from the wild in the eastern United States (Sezgin *et al.* 2004). All isothird lines had the same first and second chromosomes (*w^118^*; *VT83*). The isothird chromosomes used were *CT21*, *HFL53*, *JFL12*, *JFL29*, *MD76*, *MD80*, and *VT26*. *Idh* and *Men* enzyme activity-variant allele lines were from sets previously created using *P*-element excisions (Merritt *et al.* 2005, 2009; Lum and Merritt 2011). The *G6pd* activity-variant allele lines from these reports have been lost and were replaced with new lines generated for this project. In the *Idh* series, *IdhEx4* has wild-type IDH activity, and *IdhEx1* has no IDH activity. For *Men* activity variants, *MenEx3* has wild-type activity, and *MenEx55* has no MEN activity. *MenEx55* has a large-scale deletion spanning approximately 16 kb. This large deletion allowed us to avoid the transvection effects observed at this gene [as described in Merritt *et al.* (2005, 2009) and Lum and Merritt (2011)] and to generate heterozygotes showing approximately ∼50% MEN activity (see below). The *G6pd* activity-variant alleles were created using the *P{SUPor-P}KG05538* insertion line and the *P{Δ2-3}99B* element as a transposase source [following Merritt *et al.* (2009)]. *G6pdEx40* has wild-type G6PD activity, and *G6pdEx43* has 50% G6PD activity. Within each series, the excision chromosomes are isogenic outside of the small differences at the former location of the *P*-element caused by the *P*-element excision. To create these essentially isogeneic lines, all activity-variant alleles were placed in the same *w*; *6326*; *6326* genetic background using marker-assisted introgression, as previously described (Merritt *et al.* 2005). Line *w*; *6326*; *6326* is a subline of the isogenic line BG6326 (Hoskins *et al.* 2001). All flies were reared to adulthood at 25°, on standard cornmeal-yeast-agar-corn syrup diet, with 12-hour light/dark cycles.

### Variation across seven different third-chromosome backgrounds

We tested both the effect of varying enzyme activity and varying environmental conditions across seven different genetic backgrounds. To determine the effect of varying each of the NADP-reducing enzyme activities on the activities of the other NADP-reducing enzymes, as well as on two potential downstream phenotypes of the network (triglyceride and carbohydrate concentrations), flies with 100% and 50% activity for each of the NADP-reducing enzymes were generated. Specifically, a series of 16 paired crosses were made using the *Idh*, *G6pd*, or *Men* full-activity and knockout genotypes, as well as seven isothird-chromosome lines (second background replaced lines designated *w*;*VT83*;*i/TM8*, where *i* is isothird-chromosome 1-7). Multiple third chromosomes were used to strengthen biological significance of our results by accounting for possible variation in responses across genetic backgrounds, as previous studies have found that genetic background may affect the way in which network members interact (Merritt *et al.* 2005, 2009). We chose to vary third chromosome because both *Men* and *Idh* are located on this chromosome. For the *Men* and *Idh* lines, females from each of the seven isothird-chromosome lines were crossed with males of the excision allele lines. *G6pd* is X-linked, so to create the activity-variant genotypes in the different third chromosome backgrounds, females from the excision allele lines were crossed with males from the isothird-chromosome lines. Two bottles (50 females and 50 males per bottle) were established for each cross genotype, and flies were allowed to lay eggs for four days. To test the consistency of network interactions across varying environments, the experiment was repeated under different environmental conditions. Emerging male flies were collected, aged for five days, and exposed to one of four conditions: paraquat-induced oxidative stress, starvation stress, desiccation stress, or benign laboratory (control) conditions. Male flies were used exclusively based on findings from previous work that found similar trends in both males and females, although the trends tended to be more prominent in males (Merritt *et al.* 2009). Following treatment, flies were collected and stored at −80° until ready to be weighed, homogenized, and assayed for enzyme activities as well as triglyceride, carbohydrate, and protein concentrations.

### Oxidative stress treatment

Flies were fed 20 mM paraquat (PQ, 1,1′-Dimethyl-4,4′-bipyridinium dichloride, Sigma Aldrich, St Louis, MO, Catalog No. 856177) incorporated into a cornmeal-yeast-agar-corn syrup diet in order to simulate oxidative stress as previously described in Rzezniczak *et al.* (2011). Replicates of 25 male flies per vial were maintained for 24 hr in fly vials containing approximately 2 mL of PQ food.

### Starvation stress treatment

Flies were starved for 24 hr in vials containing 2 mL of 0.5% agar at 25° following established protocols (Marron *et al.* 2003). The agar supplied water but not nutrition.

### Desiccation stress treatment

Flies were desiccated in vials containing silica gel following established protocols (Marron *et al.* 2003). Briefly, sets of flies were placed in the bottom section of a vial that was then capped at the midway point with a commercial sponge, and approximately 4 g of silica gel (Sigma Aldrich, St Louis, MO, Catalog No. S7625) was added on top. The vial was then capped with a foam plug. Replicates of 25 male flies per vial were maintained under desiccation for 3 hr.

### Benign laboratory conditions (control)

Stress-treated flies were compared with control flies maintained under benign laboratory conditions: 25°, ambient humidity, 12-hour light/dark cycles, and standard cornmeal-yeast-agar-corn syrup diet.

### Fly weight

Flies were weighed to the nearest 0.01 mg using a Mettler Toledo microbalance MX5. Weight was used as in analyses of covariance (ANCOVA) to standardize enzyme activities, triglyceride, and carbohydrate concentrations for differences in fly size.

### Fly homogenization

Flies were homogenized in ice-cold grinding buffer (100 mM Tris-HCl, 0.15 mM NADP^+^) at a “concentration” of one fly per 100 µl of buffer and spun at 13,000 RPM for 5 min at 4° to pellet all solids. In general, assays were conducted using samples of four flies. In a few cases, insufficient flies of a specific genotype were available, and fewer flies were assayed; the homogenization buffer volume was adjusted accordingly. The supernatant was collected and vortexed, and 250 μl was added to one well of a 96-well plate. Aliquots were taken from this master plate for each of the analyses.

### Enzyme activity measurements

Activity assays were conducted on a Molecular Devices SpectraMax 384 Plus 96-well plate spectrophotometer, using 10 µl of fly homogenate and 100 µl of assay buffer (described below). Absorbance for all assays was measured at 340 nm, every 9 sec, over 3 min at 25°. Samples were assayed twice, and the means were used in further analysis. The assay buffers were previously optimized to give maximum activities (Merritt *et al.* 2005, 2009) and were as follows:

*G6PD*: 100 mM Tris-HCl, 0.32 mM NADP, 3.5mM D-glucose-6-phosphate (pH 7.4)
*IDH*: 100 mM Tris-HCl, 0.10 mM NADP, 0.84 mM MgSO_4_, 1.37 mM DL- isocitrate (pH 8.6)
*MEN*: 100 mM Tris-HCl, 0.34 mM NADP, 50 mM MnCl_2_, 50 mM malate (pH 7.4)

Enzyme activity is expressed as micromoles NADP^+^ reduced per minute per microgram soluble protein times 10,000.

### Soluble triglyceride content

Soluble triglyceride was measured using a commercially available kit (Triglyceride-SL Assay, Pointe Scientific, Canton, MI, Catalog No. T7531) following the manufacturer’s protocol. Triglycerides are a strong correlate of lipids (Clark and Keith 1988) and were quantified as a proxy for lipids. Briefly, the assays contained 10 µl homogenate and 100 µl reagent and were incubated at 37° for 10 min. Sample absorbance was measured at OD_500_ and total soluble triglyceride concentrations were determined by comparison with a commercially available standard (Pointe Scientific, Canton, MI, Catalog No. T7532). Each sample was assayed twice, and the mean used in analysis. Results are reported as micrograms of triglyceride per sample.

### Total carbohydrate content

Total carbohydrate content (*i.e.*, glycogen) was measured as previously described (Merritt *et al.* 2006). Complex carbohydrates were converted to glucose using a digestion cocktail that contained 10 µL of fly homogenate sample and 2 µL of amyloglucosidase (Sigma Aldrich, St Louis, MO, A1602) at a concentration of 1 unit/sample in 2.0 M sodium acetate buffer (pH 5.7). Samples were digested at 55° for 45 min. Following digestion, total glucose was measured using a commercially available kit (Genzyme, Cambridge, MA, Catalog No. 235-17). Digested homogenate (10 µL) was assayed in 200 µL of glucose reagent and incubated at 37° for 10 min. Sample absorbance was measured at OD_340_, and total carbohydrate concentration was determined by comparison with a glycogen standard (Sigma Aldrich, St Louis, MO, Catalog No. G0885). Results are reported as milligrams per liter.

### Soluble protein content

Soluble protein was measured by the bicinchoninic acid (BCA) assay using a commercially available kit (Pierce, Thermo Scientific, Rockford, IL, Catalog No. 23225) following the manufacturer’s protocol. Briefly, the assays contained 10 µl homogenate and 100 µl reagent and were incubated at 37° for 30 min. Sample absorbance was measured at OD_562_, and total soluble protein concentrations were determined by comparison with bovine serum albumen standards (Sigma Aldrich, St Louis, MO, A4503). Each sample was assayed twice, and the mean was used in analysis. Results are reported as milligrams per liter. Enzyme activities were standardized by soluble protein content to account for differences in size between individual flies and possible differences in the degree of homogenization between samples.

### Data analysis

All crosses were replicated in two independent bottles, with four samples taken from each bottle for each environmental condition tested. Analysis of covariance (ANCOVA) and Tukey’s honestly significant difference (HSD) multiple-comparison tests were performed using JMP 7.0 software (SAS Institute) to determine whether there were significant differences in enzyme activity or triglyceride or carbohydrate concentration between environmental conditions using protein concentration and weight as covariates. ANCOVAs were also performed to determine whether there were significant differences in NADPH enzyme activities or triglyceride or carbohydrate concentrations between the wild-type and reduced-activity genotypes for each of the four environmental conditions tested. In cases where a significant difference was found between the two genotypes, elasticity coefficients (ε) were calculated, as previously described (Merritt *et al.* 2005, 2009). Elasticity coefficients are calculated from the average of the slopes in the plots of *ln E*_2_ *vs. ln E*_1_ for each of the seven different third-chromosome backgrounds, where *E*_1_ is the enzyme activity of the gene that was genetically modified, and *E*_2_ is one of the other NADPH enzymes or triglyceride or carbohydrate concentration. In calculating elasticity coefficients, enzyme activity was expressed as nanomole NADP reduced per minute per microgram soluble protein to allow comparison with previous studies. The above analyses test whether stress conditions modify the effect of reducing NADPH enzyme activity on the NADPH network (*e.g.*, does reducing G6PD affect MEN activity under starvation), but they do not test whether the impacts of the stress conditions are significantly different (*e.g.*, does starvation have a greater impact than desiccation). To test whether the different stressors significantly differed in their impacts on the NADPH network, enzyme activity results were pooled for each genotype (*i.e.*, excision chromosome-third chromosome background pair, *e.g. w^118^*; *6326*/*VT83*; *MenEx55*/*JFL12*) under each stressor, and an ANOVA on the ratio of $\frac{50\%\;\text{genotype}}{\text{100}\%\;\text{genotype}}$ for each third-chromosome background was performed. By averaging all samples within a genotype (*i.e.*, reducing statistical power), this statistical test is a conservative estimate of the presence/absence of a difference in enzyme response across conditions.

## Results

### Response of NADPH network to stressors

**Figure 1:**
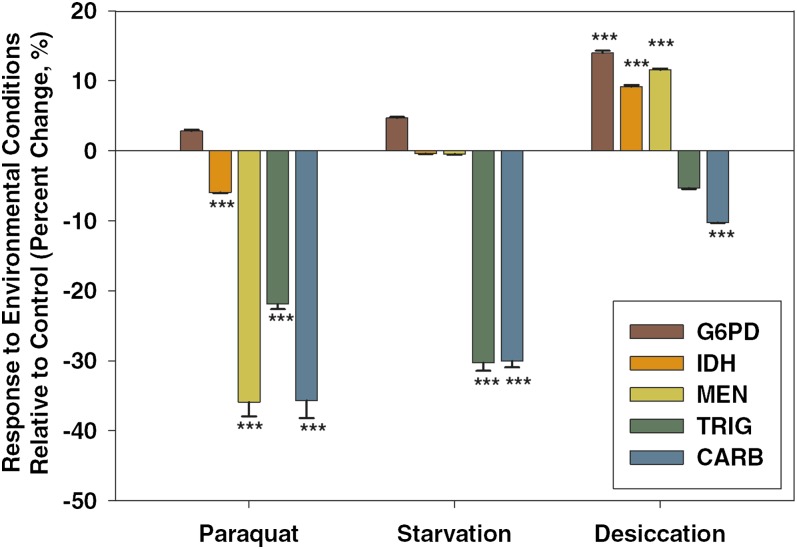
NADPH enzyme network response to environmental stressors. Cytosolic glucose-6-phosphate dehydrogenase (G6PD), cytosolic isocitrate dehydrogease (IDH), and malic enzyme (MEN) activities were measured along with triglyceride (TRIG) and carbohydrate (CARB) concentrations in wild-type flies under paraquat-induced oxidative stress, starvation, and desiccation and compared with enzyme activities measured in flies under control conditions. Activities are expressed as percentage change relative to the benign (control) conditions, and statistical significance refers to differences between stress and control. *P < 0.05, **P < 0.001, ***P < 0.0001.

Although the primary focus of this research was to examine the consistency of interactions in response to reduction in activities of the NADP-reducing enzymes, our use of environmental stressors also allowed us to quantify the response of the NADPH network to the environmental stressors themselves. The NADPH enzymes have previously been included in studies of stressors in a variety of organisms (Goodridge 1968; Kim and Park 1995; Marron *et al.* 2003; Singh *et al.* 2007), but our study allowed us to quantify interactions using a common set of methods and a uniform set of genetic backgrounds specific to this study (*G6pdEx40/i*, *IdhEx4/i*, *MenEx3/i*). Figure 1 shows the observed change in activity for G6PD, IDH, and MEN under each of the three stressors studied compared with the activities observed under benign conditions.

Enzyme activities and triglyceride and carbohydrate concentrations all varied across the three conditions, although the magnitude and direction varied between conditions (Figure 1). We found significant changes in G6PD activity across the stressors (*F*_3,671_ = 12.42, *P* < 0.0001) with increased activity in desiccated flies (Figure 1; Tukey’s HSD). IDH activity also differed significantly across the treatments (*F*_3,671_ = 17.4, *P* < 0.000): IDH activity was significantly lower under oxidative stress and higher under desiccation (Figure 1; Tukey’s HSD). Lastly, MEN activity differed significantly with treatment (*F*_3,671_ = 154.0, *P* < 0.0001), having lower activity under paraquat-induced oxidative stress but significantly higher activity under desiccation (Tukey’s HSD). The reduction in NADP-reducing enzyme activities under oxidative stress is surprising given the role of NADPH in combating oxidative stress, but it is consistent with recent work that also found reductions in these enzymes under oxidative stress generated by knocking out superoxide dismutase enzyme activity (Bernard *et al.* 2011).

Triglyceride and total carbohydrate concentrations were also measured across all four conditions (Figure 1). Triglyceride concentration was found to differ significantly with treatment (*F*_3,671_ = 61.7, *P* < 0.0001), with lower triglyceride concentrations under both oxidative stress and starvation compared with control conditions (Figure 1; Tukey’s HSD), but no significant difference under conditions of desiccation. Carbohydrate concentration also differed across treatments (*F*_3,671_ = 110.1, *P* < 0.0001), and all treatments had significantly lower carbohydrate stores compared with flies maintained under benign laboratory conditions (Tukey’s HSD). The decrease in carbohydrate, but not lipid, stores under desiccation is consistent with trends observed in previous studies (Marron *et al.* 2003) and likely reflects the fact that catabolism of carbohydrate reserves releases more water than the catabolism of lipid reserves (Schmidt-Nielsen 1997).

### Responses to variation in G6PD activity

**Figure 2:**
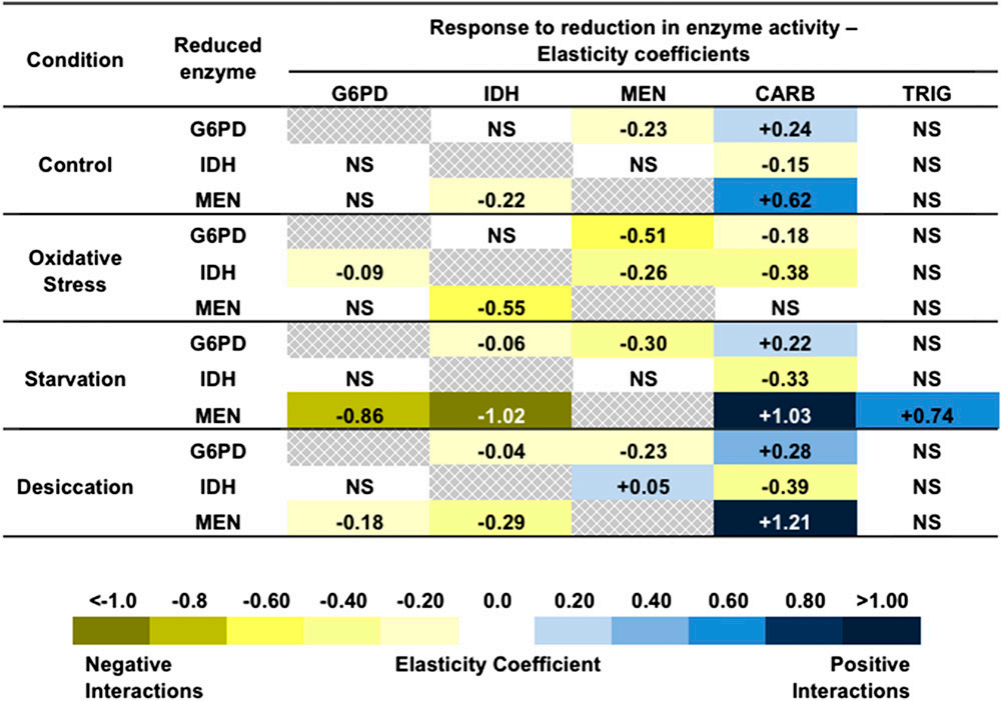
Elasticity coefficients for reductions in glucose-6-phosphate dehydrogenase (G6PD), isocitrate dehydrogenase (IDH), and malic enzyme (MEN) across conditions. Yellow represents negative interactions, and blue represents positive interactions. The shading of the cells represents the magnitude of the interactions with darker shading representing interactions of a greater magnitude. CARB, total carbohydrate concentration; NS, not significant; TRIG, triglyceride concentration.

Reduced G6PD-activity flies (males with the *G6pdEx43* allele) averaged approximately 41.8 ± 3.04% of the G6PD activity of wild-type flies (males with the *G6pdEx40* allele) across all three stress treatments and controls. Elasticity coefficients and statistics are summarized in Figure 2. The elasticity coefficients with associated errors and statistics can be found in Supporting Information, Table S1.

**Figure 3:**
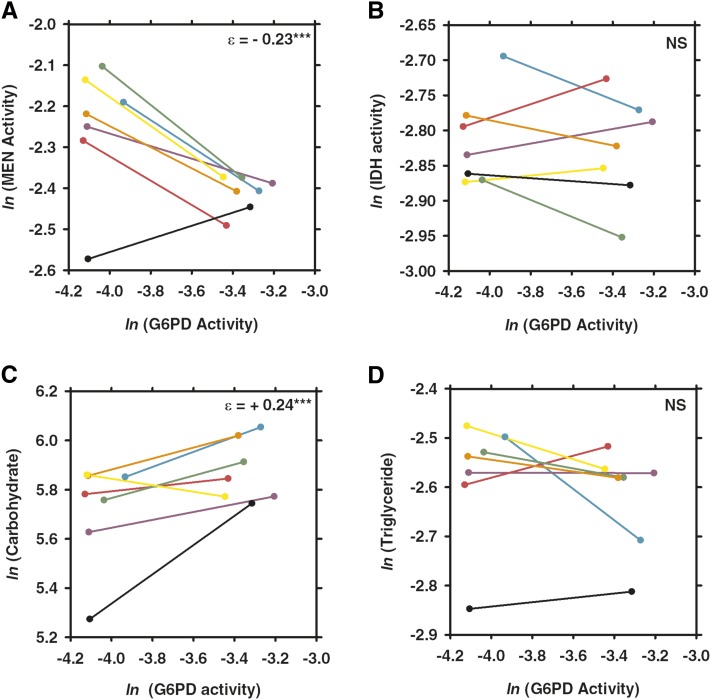
Comparison of the effects of changes in glucose-6-phosphate dehydrogenase (G6PD) activity across seven different third chromosomes under control conditions. Two synthetic *G6pd* alleles, one with wild-type activity and one with 50% activity, were crossed to seven different third chromosomes. Each pair of points connected by a line shows the difference in (A) malic enzyme (MEN) activity, (B) isocitrate dehydrogenase (IDH) activity, (C) carbohydrate, or (D) triglyceride concentrations in a different third-chromosome background, with different backgrounds being represented by different colors (CT21, teal; HFL53, purple; JFL12, pink; JFL29, green; MD76, orange; MD80, yellow; VT26, black). Values from the full activity flies are on the right end of the line and those from the partial activity flies are on the left end of the line. Enzyme activity units are the natural log (*ln*) of 10× nmol NADP reduced/min · µg protein. Carbohydrate concentration units are in mg/L, and triglyceride concentration units are in mmol/L. NS, not significant. *P < 0.05; **P < 0.001, ***P < 0.0001.

**Figure 4:**
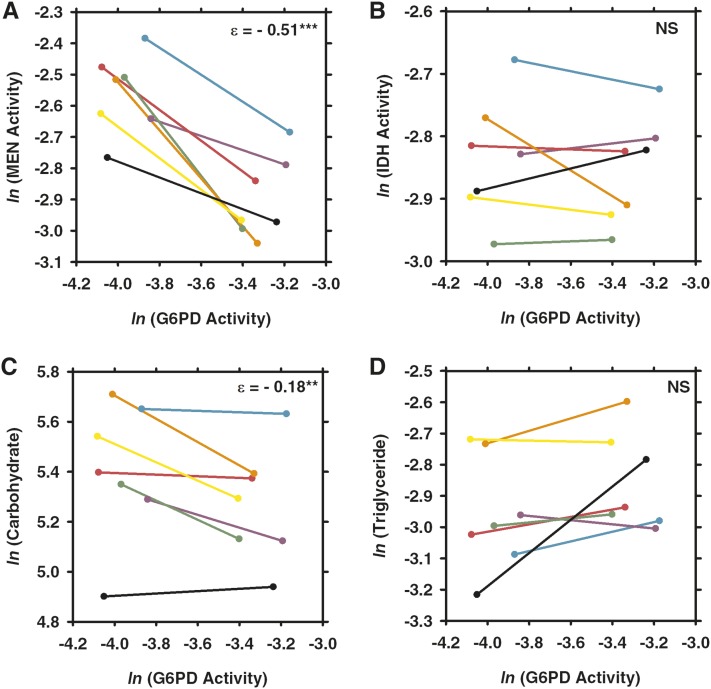
Comparison of the effects of changes in G6PD activity across seven different third chromosomes under paraquat-induced oxidative stress. Flies with one of two synthetic *G6pd* alleles, one with wild-type activity and one with 50% activity, were crossed to seven different third chromosomes and were fed paraquat for 24 hr to induce oxidative stress and then assayed for (A) malic enzyme (MEN) activity, (B) isocitrate dehydrogenase (IDH) activity, (C) carbohydrate concentration, or (D) triglyceride concentration. Each color represents a different third chromosome (see Figure 3). Values from the full activity flies are on the right end of the line and those from the partial activity flies are on the left end of the line. Abbreviations, values, and statistical significance are as in Figure 3.

**Figure 5:**
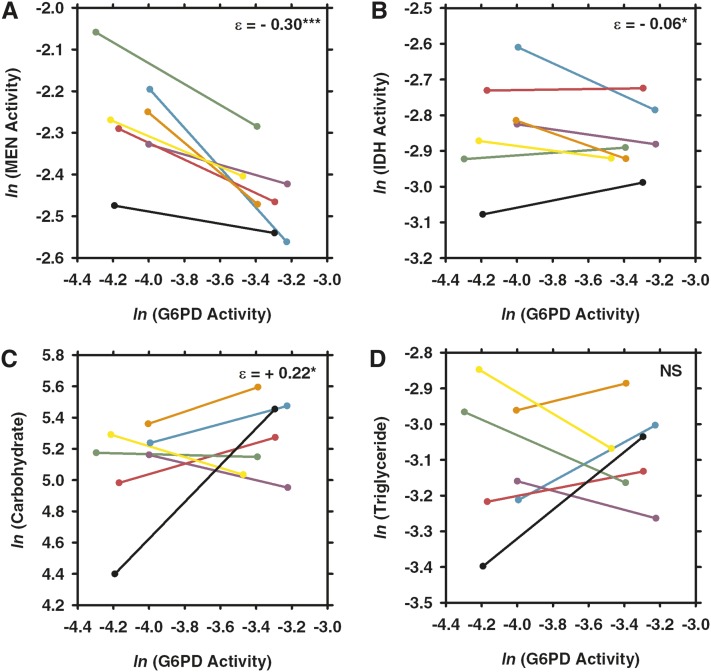
Comparison of the effects of changes in G6PD activity across seven different third chromosomes under starvation. Two synthetic *G6pd* alleles, one with wild-type activity and one with 50% activity, were crossed to seven different third chromosomes and starved for 24 hr. Each pair of points connected by a line shows the difference in (A) MEN activity, (B) IDH activity, (C) carbohydrate, or (D) triglyceride concentration in a different third-chromosome background, each represented by a different color (see Figure 3). Differences in responses represent the impact of starvation stress on interactions within the NADPH enzyme network. Abbreviations, values, and statistical significance are as in Figure 3.

**Figure 6:**
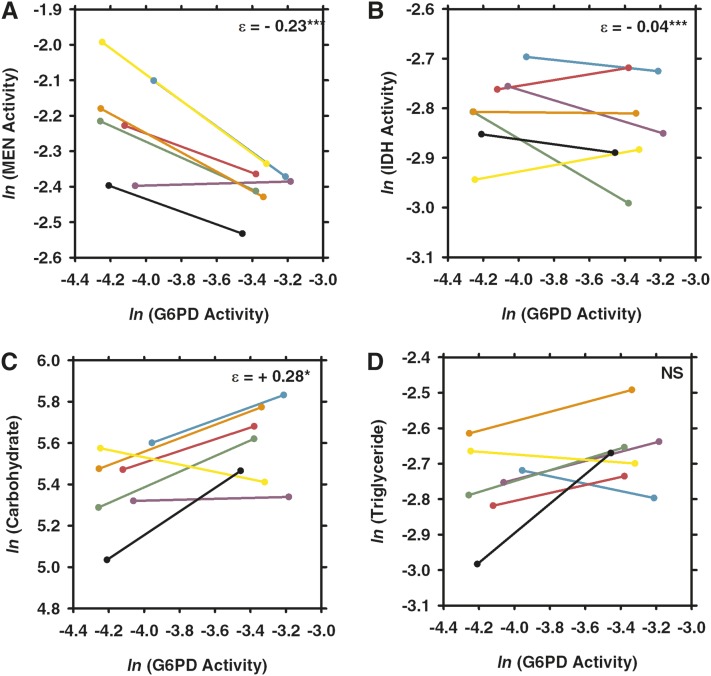
Comparison of the effects of changes in G6PD activity across seven different third chromosomes under desiccation. Two synthetic *G6pd* alleles, one with wild-type activity and one with 50% activity, were crossed to seven different third chromosomes and maintained under low humidity and the absence of food or water for 3 hr and then assayed for (A) MEN activity, (B) IDH activity, (C) carbohydrate concentration, and (D) triglyceride concentration. Each color represents a different third chromosome (see Figure 3). Values from the full activity flies are on the right end of the line, and those from the partial activity flies are on the left end of the line. Abbreviations, values, and statistical significance are as in Figure 3.

MEN activity varied with the engineered variation in G6PD activity under control conditions (see Figures 2 and 3A) as well as under each of the three stressors (Figures 4A, 5A, and 6A). Under all conditions, the changes in MEN activity were compensatory (*i.e.*, the decrease in G6PD activity resulted in an increase of MEN activity), consistent with previous studies that suggested that the NADP-reducing enzymes work concurrently to maintain a cellular pool of NADPH (Merritt *et al.* 2005, 2009). The responses in MEN activity across the different conditions were found to be significantly different (*F*_3,27_ = 6.6, *P* < 0.0033); the response in MEN activity under oxidative stress was significantly larger than that under any other condition (Tukey’s HSD). This significantly larger response (*i.e.*, increase in MEN activity) suggests that this condition generates the greatest need for NADPH, consistent with the substantial role for G6PD in the reduction of NADP^+^ under oxidative stress proposed by others (Pandolfi *et al.* 1995; Pollak *et al.* 2007; Ying 2008; Agledal *et al.* 2010).

Like MEN activity, IDH activity varied in response to the reduction in G6PD under the tested environmental conditions, but responses varied (*i.e.*, both increases and decreases in activity of varying magnitudes were observed) across genetic backgrounds. IDH activity was significantly higher in the low G6PD activity flies under conditions of starvation and desiccation, although the differences were small: +5.3% and +3.3%, respectively (Figures 2, 5B, and 6B; Table S1). Directly measuring enzyme activity enables us to accurately quantify such small differences, but it is unclear what the biological impact of such small changes in enzyme activity is. There was no statistically significant response in IDH activity to the reduction in G6PD activity under control or oxidative stress conditions (Figures 2, 3B, and 4B; Table S1), although some of the genetic backgrounds did show small compensatory increases in IDH activity, and others showed decreases in activity. This heterogeneity in response stresses the importance of examining genetic interactions across multiple genetic backgrounds, it and also suggests that, although no statistical change was observed overall, the change in G6PD activity has an effect on IDH activity even if the effect is small and not consistent across genetic backgrounds. Statistical tests examining the responses in IDH activity across environmental conditions did not find any significant differences, suggesting that overall IDH response to reduction in G6PD remains fairly consistent across conditions.

Carbohydrate stores (*i.e.*, glycogen stores) were found to vary with reduced G6PD activity across all conditions (Figure 2; Table S1). In particular, carbohydrate stores were significantly lower in the low G6PD activity flies under control, starvation, and desiccation conditions (Figures 2, 3C, 5C, and 6C; Table S1). These reductions in carbohydrate concentration were fairly consistent, ranging from decreases of 11.3–17.6%. Surprisingly, under paraquat-induced oxidative stress, carbohydrate stores were found to be 13.2% higher in the low G6PD activity flies (Figures 2 and 4C; Table S1). The responses in carbohydrate concentration were found to differ between conditions (*F*_3,27_ = 5.8 *P* < 0.0059); the carbohydrate concentration under oxidative stress differed from all conditions except starvation (Tukey’s HSD).

Overall, there was no significant change in triglyceride concentrations in response to the change in G6PD activity under any of the tested conditions. Nevertheless, in certain genetic backgrounds, there were differences in triglyceride concentration between the two *G6pd* genotypes (Figures 3D, 4D, 5D, and 6D; Table S1). For example, under starvation, four of the seven genetic backgrounds show fairly large decreases in triglyceride concentration, whereas the remaining three show large increases in concentration (Figure 5D; Table S1). This type of variability between genetic backgrounds is consistent with triglyceride being a complex downstream phenotype of the NADPH enzyme network with a number of factors outside of the network influencing the concentration, and it is consistent with previous studies (Merritt *et al.* 2005, 2009).

### Responses to variation in IDH activity

IDH activity in the reduced-activity flies (*IdhEx1/i* heterozygotes) averaged 45.9 ± 0.47% of that of the full-activity flies (*IdhEx4/i* heterozygotes) across all treatments. Responses to lowered IDH activity were analyzed identically to that of reduced G6PD activity, described above. Elasticity coefficients are summarized in Figure 2, but elasticity coefficient figures are not shown. The elasticity coefficients with associated errors and statistics can be found in Table S2.

G6PD activity was found to change in response to the reduction in IDH activity only under a single condition: oxidative stress. Under oxidative stress, G6PD activity was 6.3% higher in the low IDH flies than the wild-type IDH flies (Figure 2; Table S2). There was no significant change in G6PD activity in response to the reduction in IDH under any of the other conditions (Figure 2; Table S2), and when we compared the responses across conditions, the small compensatory increase in G6PD activity under oxidative stress did not significantly differ from the other conditions in which no response was observed. The lack of a response in G6PD activity under benign conditions is consistent with the findings of previous studies (Merritt *et al.* 2009). Overall, our results show little interaction between G6PD and changes in IDH activity.

In response to the reduction in IDH activity, the magnitude and directionality of MEN activity changed depending on the environmental condition. As expected, under conditions of oxidative stress, there was a significant increase in MEN activity in the low IDH activity flies (Figure 2; Table S2). This compensatory increase is consistent with IDH playing an important role in providing NADPH under conditions of oxidative stress as documented in previous studies (Lee *et al.* 2002). On the other hand, under conditions of desiccation, there was a small but significant decrease in MEN activity in the reduced IDH flies (Figure 2; Table S2). This decrease counters an expectation of NADPH compensation, but it is consistent with previous studies (Bentley *et al.* 1983; Merritt *et al.* 2009). There was no significant change observed in the activity of MEN under control or starvation conditions; however, the observed trend in both cases was a small decrease in activity. It is likely that the significant decrease in MEN activity observed under desiccation is an amplification of the trend observed under control and starvation. When comparing across conditions, the difference in MEN activity between the wild-type and low IDH activity flies was statistically different under conditions of oxidative stress (*F*_3,27_ = 8.1, *P* < 0.0013, Tukey’s HSD test).

Interestingly, carbohydrate concentration was increased in the low IDH activity flies across all conditions: increases ranged from 7.9 to 36.0% (Figure 2; Table S2) across the environmental conditions, and were all significantly different from concentrations in wild-type flies; however, none of the increases is significantly different across conditions (*i.e.*, a reduction in IDH activity is accompanied by an increase in carbohydrate concentration regardless of the environment). This uniform increase in carbohydrate concentration was not observed in response to a decrease in G6PD or MEN (described below) in which reductions in carbohydrates were observed. In contrast to carbohydrates, no significant differences were observed in triglyceride concentration between the two IDH activity groups under any of the environmental conditions (Figure 2; Table S2), suggesting the changes in carbohydrate concentrations are not a general feature of metabolism for energy storage. Instead, this carbohydrate increase may indicate that the decrease in IDH activity may cause metabolic dysregulation.

### Responses to variation in MEN activity

The reduced MEN activity flies (*MenEx55*/*i* heterozygotes) averaged 68.8 ± 2.12% the MEN activity of the full activity flies (*MenEx3*/*i* heterozygotes). Responses to lowered IDH activity were analyzed identically to the responses to reduced G6PD and IDH activity described above. Elasticity coefficients are summarized in Figure 2, but elasticity coefficient figures are not shown. The elasticity coefficients with associated errors and statistics can be found in Table S3.

IDH activity was significantly higher in the lower MEN activity flies under all tested environmental conditions, in apparent compensation for the decrease in MEN activity (Figure 2; Table S3). The increase in IDH activity varied from 9.5% to 18.8% across conditions, but the amount of IDH increase was not significantly different across these conditions (*i.e.*, the amount of apparent compensation in IDH activity did not vary with environmental conditions).

G6PD activity was only significantly different in the low MEN flies under conditions of starvation and desiccation: under starvation, G6PD activity was 12.1% higher in the low MEN flies, and under desiccation, G6PD was 5.1% higher in the low MEN flies (Figure 2; Table S3). There was no change in G6PD activity in response to the variation in MEN activity under benign or oxidative stress conditions. This apparent difference in response suggests that MEN may be a larger contributor of NADPH under conditions of starvation, but the different responses in G6PD activity were not significantly different across environmental conditions.

Carbohydrate concentrations were lower in the low MEN activity flies under all conditions except oxidative stress in which no change was observed. The reductions in carbohydrate stores in response to the reduction in MEN activity ranged from 15.8% to 34.9% (Figure 2; Table S3) and were significantly different across conditions (*F*_3,27_ = 10.3, *P* < 0.0004). Specifically, the reduction in carbohydrate concentration between *Men* genotypes under starvation was statistically greater than what was observed under control conditions (Tukey’s HSD test). Although the lack of a change in carbohydrates under oxidative stress was not found to differ from control conditions (in which the reduction in carbohydrates was lowest), the change in carbohydrates under oxidative stress did differ from starvation and desiccation (in which the reduction in carbohydrates was greater). The trend of the response in carbohydrate stores in response to a reduction in MEN is similar to observations made in response to the reduction in G6PD; carbohydrates decreased under every condition except oxidative stress.

In contrast to the change in carbohydrates and consistent with earlier work (Merritt *et al.* 2005, 2009), there was little change in triglyceride concentration with changes in MEN activity or environment. The only significant change in triglyceride concentration was observed under conditions of starvation. In this case, triglyceride concentration was 14.4% lower in the low MEN activity flies (Figure 2; Table S3). Statistical tests indicated that the response in triglyceride concentrations varied across conditions (*F*_3,27_ = 13.5, *P* < 0.0001), and the change in triglyceride under starvation differed from that observed under control and oxidative stress conditions (Tukey’s HSD).

## Discussion

We examined a simple metabolic network to directly quantify the consistency of genetic interactions across varying environmental conditions and found that interactions between network members change both in directionality and magnitude under different environmental conditions. Overall, this study emphasizes the importance of moving away from mapping biological networks as static snapshots; using multiple conditions and genetic backgrounds can uncover new, and previously undetected, interactions.

### NADP-reducing enzyme responses to reduction in G6PD activity

A combination of reduced G6PD activity and oxidative stress was associated with a larger increase in MEN than that observed under any other conditions. Previous work under benign conditions had found little impact of variation in G6PD, suggesting only a minor role for G6PD in NADPH generation (Merritt *et al.* 2009). The connection between G6PD and MEN observed here suggests that G6PD activity generates a substantial amount of NADPH under conditions of oxidative stress, consistent with previous studies that found G6PD activity to be important under these conditions in other systems (Pandolfi *et al.* 1995; Ursini *et al.* 1997; Salvemini *et al.* 1999; Singh *et al.* 2007; Legan *et al.* 2008). A role for G6PD as a major contributor of NADPH under oxidative stress is consistent with proposed metabolic rerouting to the pentose phosphate pathway (PPP) under conditions of oxidative stress (Ralser *et al.* 2007). Such a model of metabolic rerouting is also supported in our results by the observed reductions in MEN and IDH and by the lack of change in G6PD activity in wild-type flies in response to oxidative stress (Figure 1). It appears that funneling available glucose to the PPP results in generating NADPH to combat ROS through the actions of G6PD (and 6PGD), while flux through glycolysis is downregulated, thereby avoiding further ROS production by electron leaking in the electron transport chain (Grüning *et al.* 2011).

### NADP-reducing enzyme responses to reduction in IDH activity

Perhaps the most striking variation in interaction that we observed was the relationship between MEN and IDH driven by reduction in IDH activity: varying environmental conditions not only changed the magnitude but also the direction of the interaction. Under benign conditions, starvation, and desiccation, MEN activity was lower in low IDH activity flies (*i.e.*, reduction in IDH activity was accompanied by a parallel reduction in MEN activity). The parallel reduction suggests some form of co-regulation of MEN and IDH, but not in a compensatory fashion. The parallel decrease was only statistically significant under desiccation; however, the pattern was visible under other conditions (*i.e.*, control and starvation) and is consistent with previous findings (Bentley *et al.* 1983; Merritt *et al.* 2009). In contrast, under conditions of oxidative stress, we observed significantly higher MEN activity in the low IDH activity flies (*i.e.*, compensatory increase in MEN activity in response to lower IDH activity). First, this finding indicates that although G6PD is a major contributor of NADPH under oxidative stress (above), IDH also plays an important role in generating NADPH under these conditions, consistent with findings of previous studies (Lee *et al.* 2002; Singh *et al.* 2007). In addition, the parallel reduction in MEN and IDH activities seen under most conditions may be due to a coupled role for these two enzymes in certain types of pyruvate-shuttling mechanisms but not in others (Pongratz *et al.* 2007; Guay *et al.* 2007). More specifically, our data suggest that, under oxidative stress, there is a shift from MEN and IDH working in tandem to MEN functioning independently of IDH. This pattern is consistent with increased cellular demand for NADPH under oxidative stress driving this change in the directionality of the interaction, and it suggests future research to examine other conditions in which NADPH is in high demand.

### NADP-reducing enzyme responses to reduction in MEN activity

Interactions in response to a reduction in MEN remained relatively consistent across environmental conditions. There did appear to be a trend for greater compensation in both G6PD and IDH under conditions of starvation, suggesting an elevated need for MEN activity under this condition, consistent with a change in the enzymatic source of NADPH in response to glucose availability (Mailloux and Harper 2010). These interactions suggest that NADPH continues to be an important cofactor for cellular processes during starvation, despite the fact that lipogenesis is downregulated. Interestingly, several studies have found that starvation can induce a certain level of oxidative stress (Scherz-Shouval *et al.* 2007; Chen *et al.* 2009), likely due to an upregulation of peroxisomal β-oxidation (Van den Branden *et al.* 1984). An increase in ROS formation suggests that, under starvation, the NADPH generated (primarily by MEN) is used in antioxidation. This finding, suggesting an important role for NADPH in antioxidation under starvation, is consistent with a study by Minard and Mcalister-Henn (1999) that found that the NADP-reducing enzymes were required in yeast to detoxify the H_2_O_2_ generated by peroxisomal β-oxidation.

### Carbohydrate and triglyceride response to reduction in NADP-reducing enzymes

Interactions within the NADPH enzyme network are not restricted to the enzymes; we also found that changes in network member interactions affected downstream phenotypes. In particular, we examined carbohydrate (*i.e.*, glycogen) and lipid content (via triglycerides), two complex phenotypes linked to metabolic enzyme activity and NADPH concentrations (Geer *et al.* 1979; Geer and Laurie-Ahlberg 1984). We found that perturbations in the NADPH network heavily influence carbohydrate content, a phenotype thought to be regulated, at least in part, by insulin signaling (Rulifson *et al.* 2002). This apparent connection of glycogen content and the NADP-reducing enzymes suggests that these enzymes play a role in *Drosophila* insulin signaling. Specifically, we observed that reductions in IDH activity resulted in increases in glycogen content across all environmental conditions mimicking ablation of insulin producing cells (IPC) in *Drosophila melanogaster*, which, surprisingly, also showed a decrease in glycogen content (Haselton *et al.* 2010). In light of the results of the study by Haselton *et al.* (2010), our results suggest that decreased IDH activity may augment insulin generation or secretion. A role for IDH in insulin signaling is consistent with studies in pancreatic β-cells where blockage of cytosolic IDH impairs insulin secretion (Ronnebaum *et al.* 2006). On the other hand, reductions of MEN and G6PD activity showed very different responses in glycogen concentrations with parallel reductions in glycogen levels under all conditions except oxidative stress. This differential effect of reduced activity between the enzymes does suggest that the mechanism by which these enzymes affect insulin signaling cannot be fully attributed to changes in NADPH concentration. Future work will further examine the connection between NADP-reducing enzymes and insulin secretion in *D. melanogaster*.

In contrast to the extensive changes in glycogen content, changes in total lipid were observed only under a single condition: lipids decreased in the reduced *Men* genotype starvation. Apparently, even with the compensation seen in both G6PD and IDH activity in response to the reduction in MEN under starvation, NADPH availability for any basal lipogenesis is decreased under these conditions. This reduction is consistent with MEN being the major contributor of NADPH under conditions of starvation.

### Genetic background effects

In almost all cases, we observed differences in the responses of the enzymes and metabolite concentrations when these were quantified in different genetic backgrounds (*e.g.*, see Figures 3–6). As expected, these results indicate that the interactions of the NADP-reducing enzymes are influenced by other factors, such as concentrations of gene products that may or may not *directly* act on the network. Although we did not identify the specific differences between the genetic backgrounds, the variation in the third chromosome is likely a reasonable estimate of the type of genetic variation present in wild populations. Often studies take a simplified approach by focusing on a specific mutation in a specific genetic background and do not consider that phenotypic variability can be found when a mutation is studied in different backgrounds. The pronounced variations in responses across backgrounds that we demonstrate suggest that such studies in a single background should be interpreted with caution.

### Conclusion

This study provides evidence that interactions between members of the NADPH enzyme network can change, both in directionality and magnitude, in response to differing environmental conditions. This conclusion is consistent with studies of other, larger-scale genetic networks (Bandyopadhyay *et al.* 2010) and emphasizes the importance of examining networks across multiple environmental conditions. Although the environmental conditions used in this study were artificially generated in the lab, they do mimic the conditions of stress that animals encounter in the wild and are biologically relevant for this system. Oxidative stress, for example, can be caused by ageing or exposures to ultraviolet light, ionizing radiation, and environmental toxins [reviewed in Finkel and Holbrook (2000)]. It is likely that changes in network interactions, which are important for animals to survive stress in the wild, affect the evolution and function of network traits. To fully understand network functions, research should consider the effect of changing external stimuli.

## Supplementary Material

Supporting Information
