# Supplementary material for: Interactions of NADP-Reducing Enzymes Across Varying Environmental Conditions: A Model of Biological Complexity
Source: G3 (Bethesda). 2012 Dec 1;2(12):1613–23. doi: 10.1534/g3.112.003715 (PMC3516483; doi:10.1534/g3.112.003715)
Supplement: Supporting Information [file supp_2_12_1613__index.html]

Supporting Information 

# Interactions of NADP-Reducing Enzymes Across Varying Environmental Conditions: A Model of Biological Complexity

## Supporting Information for Rzezniczak and Merritt, 2012

**Files in this Data Supplement:**

- Supporting Information - Table S1-S3 (PDF, 69 KB)
- Table S1 - Response to 50% reduction in glucose-6-phosphate dehydrogenase (G6PD) activity (PDF, 57 KB)
- Table S2 - Response to 50% reduction in isocitrate dehydrogenase (IDH) activity (PDF, 57 KB)
- Table S3 - Response to 50% reduction in malic enzyme (MEN) activity (PDF, 58 KB)
