# Supplementary material for: Interactions of NADP-Reducing Enzymes Across Varying Environmental Conditions: A Model of Biological Complexity
Source: G3 (Bethesda). 2012 Dec 1;2(12):1613–23. doi: 10.1534/g3.112.003715 (PMC3516483; doi:10.1534/g3.112.003715)
Supplement: Supporting Information [file supp_2.12.1613_003715SI.pdf]

Table S1 Response to 50% reduction in glucose-6-phosphate dehydrogenase (G6PD) activity

Environmental Condition	Enzyme/ Metabolite	Percent Difference	Elasticity Coefficient	*F*-ratio From ANCOVA[a]
Control	IDH	NS	NS	NS
	MEN	+17.9%	-0.23 ± 0.072	F = 47.0, P < 0.0001
	CARB	-11.3%	+0.24 ± 0.085	F = 28.6, P < 0.0001
	TRIG	NS	NS	NS
Oxidative Stress	IDH	NS	NS	NS
	MEN	+40.0%	-0.51 ± 0.090	F = 89.6, P < 0.0001
	CARB	+13.2%	-0.18 ± 0.057	F = 10.2, P < 0.0019
	TRIG	NS	NS	NS
Starvation	IDH	+5.3%	-0.06 ± 0.046	F = 7.9, P < 0.0059
	MEN	+21.8%	-0.30 ± 0.093	F = 44.1, P < 0.0001
	CARB	-16.1%	+0.22 ± 0.021	F = 4.8, P < 0.0304
	TRIG	NS	NS	NS
Desiccation	IDH	+3.3%	-0.04 ± 0.036	F = 5.2, P < 0.0239
	MEN	+23.4%	-0.23 ± 0.050	F = 40.6, P < 0.0001
	CARB	-17.6%	+0.28 ± 0.097	F = 7.6, P < 0.0070
	TRIG	NS	NS	NS

a. Degrees of freedom for F ratio: $F_{1,111}$

Abbreviations: IDH – Isocitrate dehydrogenase, MEN – Malic enzyme, CARB –Total carbohydrate concentration, TRIG – Triglyceride concentration, NS – Not significant

Table S2 Response to 50% reduction in isocitrate dehydrogenase (IDH) activity

Environmental Condition	Enzyme/ Metabolite	Percent Difference	Elasticity Coefficient	F-ratio From ANCOVA[a]
Control	G6PD	NS	NS	NS
	MEN	NS	NS	NS
	CARB	+7.9%	-0.15 ± 0.066	*F* = 8.6, *P* < 0.0042
	TRIG	NS	NS	NS
Oxidative Stress	G6PD	+6.3%	-0.09 ± 0.046	*F* = 12.9, *P* < 0.0005
	MEN	+21.2%	-0.26 ± 0.057	*F* = 36.4, *P* < 0.0001
	CARB	+28.4%	-0.38 ± 0.084	*F* = 32.3, *P* < 0.0001
	TRIG	NS	NS	NS
Starvation	G6PD	NS	NS	NS
	MEN	NS	NS	NS
	CARB	+25.3%	-0.33 ± 0.082	*F* = 36.6, *P* < 0.0001
	TRIG	NS	NS	NS
Desiccation	G6PD	NS	NS	NS
	MEN	-6.6%	+0.05 ± 0.055	*F* = 10.4, *P* < 0.0017
	CARB	+36.0%	-0.39 ± 0.057	*F* = 45.4, P < 0.0001
	TRIG	NS	NS	NS

a. Degrees of freedom for *F* ratio: $F_{1,111}$

Abbreviations: G6PD – Glucose-6-phosphate dehydrogenase, MEN – Malic enzyme, CARB – Total carbohydrate concentration, TRIG – Triglyceride concentration, NS – Not significant

Table S3 Response to 50% reduction in malic enzyme (MEN) activity

Environmental Condition	Enzyme/ Metabolite	Percent Difference	Elasticity Coefficient	F-ratio From ANCOVA[a]
Control	G6PD	NS	NS	NS
	IDH	+9.5%	-0.22 ± 0.060	F = 11.1, P < 0.0012
	CARB	-15.8%	+0.62 ± 0.096	F = 24.2, P < 0.0001
	TRIG	NS	NS	NS
Oxidative Stress	G6PD	NS	NS	NS
	IDH	+15.9%	-0.50 ± 0.163	F = 34.0, P < 0.0001
	CARB	NS	NS	NS
	TRIG	NS	NS	NS
Starvation	G6PD	+12.1%	-0.86 ± 0.557	F = 9.7, P < 0.0024
	IDH	+18.8%	-1.02 ± 0.431	F = 10.1, P < 0.0020
	CARB	-34.9%	+1.03 ± 0.200	F = 35.4, P < 0.0001
	TRIG	-14.4%	+0.74 ± 0.164	F = 6.0, P < 0.0158
Desiccation	G6PD	+5.1%	-0.18 ± 0.092	F = 7.1, P < 0.0087
	IDH	+9.5%	-0.29 ± 0.132	F = 66.0, P < 0.0001
	CARB	-21.7%	+1.21 ± 0.203	F = 57.3, P < 0.0001
	TRIG	NS	NS	NS

a. Degrees of freedom for *F* ratio: $F_{1,111}$

Abbreviations: G6PD – Glucose-6-phosphate dehydrogenase, IDH – Isocitrate dehydrogenase, CARB – Total carbohydrate concentration, TRIG – Triglyceride concentration, NS – Not significant
