# Supplementary material for: Interactions of NADP-Reducing Enzymes Across Varying Environmental Conditions: A Model of Biological Complexity
Source: G3 (Bethesda). 2012 Dec 1;2(12):1613–23. doi: 10.1534/g3.112.003715 (PMC3516483; doi:10.1534/g3.112.003715)
Supplement: Supporting Information [file supp_2.12.1613_TableS1.pdf]

Table S1 Response to 50% reduction in glucose-6-phosphate dehydrogenase (G6PD) activity

Environmental Condition	Enzyme/ Metabolite	Percent Difference	Elasticity Coefficient	*F*-ratio From ANCOVA[a]
Control	IDH	NS	NS	NS
	MEN	+17.9%	-0.23 ± 0.072	$F = 47.0$, $P < 0.0001$
	CARB	-11.3%	+0.24 ± 0.085	$F = 28.6$, $P < 0.0001$
	TRIG	NS	NS	NS
Oxidative Stress	IDH	NS	NS	NS
	MEN	+40.0%	-0.51 ± 0.090	$F = 89.6$, $P < 0.0001$
	CARB	+13.2%	-0.18 ± 0.057	$F = 10.2$, $P < 0.0019$
	TRIG	NS	NS	NS
Starvation	IDH	+5.3%	-0.06 ± 0.046	$F = 7.9$, $P < 0.0059$
	MEN	+21.8%	-0.30 ± 0.093	$F = 44.1$, $P < 0.0001$
	CARB	-16.1%	+0.22 ± 0.021	$F = 4.8$, $P < 0.0304$
	TRIG	NS	NS	NS
Desiccation	IDH	+3.3%	-0.04 ± 0.036	$F = 5.2$, $P < 0.0239$
	MEN	+23.4%	-0.23 ± 0.050	$F = 40.6$, $P < 0.0001$
	CARB	-17.6%	+0.28 ± 0.097	$F = 7.6$, $P < 0.0070$
	TRIG	NS	NS	NS

a. Degrees of freedom for *F* ratio: $F_{1,111}$

Abbreviations: IDH – Isocitrate dehydrogenase, MEN – Malic enzyme, CARB –Total carbohydrate concentration, TRIG – Triglyceride concentration, NS – Not significant
