# Supplementary material for: Interactions of NADP-Reducing Enzymes Across Varying Environmental Conditions: A Model of Biological Complexity
Source: G3 (Bethesda). 2012 Dec 1;2(12):1613–23. doi: 10.1534/g3.112.003715 (PMC3516483; doi:10.1534/g3.112.003715)
Supplement: Supporting Information [file supp_2.12.1613_TableS2.pdf]

Table S2 Response to 50% reduction in isocitrate dehydrogenase (IDH) activity

Environmental Condition	Enzyme/ Metabolite	Percent Difference	Elasticity Coefficient	F-ratio From ANCOVA[a]
Control	G6PD	NS	NS	NS
	MEN	NS	NS	NS
	CARB	+7.9%	-0.15 ± 0.066	F = 8.6, P < 0.0042
	TRIG	NS	NS	NS
Oxidative Stress	G6PD	+6.3%	-0.09 ± 0.046	F = 12.9, P < 0.0005
	MEN	+21.2%	-0.26 ± 0.057	F = 36.4, P < 0.0001
	CARB	+28.4%	-0.38 ± 0.084	F = 32.3, P < 0.0001
	TRIG	NS	NS	NS
Starvation	G6PD	NS	NS	NS
	MEN	NS	NS	NS
	CARB	+25.3%	-0.33 ± 0.082	F = 36.6, P < 0.0001
	TRIG	NS	NS	NS
Desiccation	G6PD	NS	NS	NS
	MEN	-6.6%	+0.05 ± 0.055	F = 10.4, P < 0.0017
	CARB	+36.0%	-0.39 ± 0.057	F = 45.4, P < 0.0001
	TRIG	NS	NS	NS

a. Degrees of freedom for F ratio: $F_{1,111}$

Abbreviations: G6PD – Glucose-6-phosphate dehydrogenase, MEN – Malic enzyme, CARB – Total carbohydrate concentration, TRIG – Triglyceride concentration, NS – Not significant
