# Supplementary material for: Interactions of NADP-Reducing Enzymes Across Varying Environmental Conditions: A Model of Biological Complexity
Source: G3 (Bethesda). 2012 Dec 1;2(12):1613–23. doi: 10.1534/g3.112.003715 (PMC3516483; doi:10.1534/g3.112.003715)
Supplement: Supporting Information [file supp_2.12.1613_TableS3.pdf]

Table S3 Response to 50% reduction in malic enzyme (MEN) activity

Environmental Condition	Enzyme/ Metabolite	Percent Difference	Elasticity Coefficient	F-ratio From ANCOVA[a]
Control	G6PD	NS	NS	NS
	IDH	+9.5%	-0.22 ± 0.060	F = 11.1, P < 0.0012
	CARB	-15.8%	+0.62 ± 0.096	F = 24.2, P < 0.0001
	TRIG	NS	NS	NS
Oxidative Stress	G6PD	NS	NS	NS
	IDH	+15.9%	-0.50 ± 0.163	F = 34.0, P < 0.0001
	CARB	NS	NS	NS
	TRIG	NS	NS	NS
Starvation	G6PD	+12.1%	-0.86 ± 0.557	F = 9.7, P < 0.0024
	IDH	+18.8%	-1.02 ± 0.431	F = 10.1, P < 0.0020
	CARB	-34.9%	+1.03 ± 0.200	F = 35.4, P < 0.0001
	TRIG	-14.4%	+0.74 ± 0.164	F = 6.0, P < 0.0158
Desiccation	G6PD	+5.1%	-0.18 ± 0.092	F = 7.1, P < 0.0087
	IDH	+9.5%	-0.29 ± 0.132	F = 66.0, P < 0.0001
	CARB	-21.7%	+1.21 ± 0.203	F = 57.3, P < 0.0001
	TRIG	NS	NS	NS

a. Degrees of freedom for F ratio: $F_{1,111}$

Abbreviations: G6PD – Glucose-6-phosphate dehydrogenase, IDH – Isocitrate dehydrogenase, CARB – Total carbohydrate concentration, TRIG – Triglyceride concentration, NS – Not significant
